# Analysis of the impact of COVID-19 on Scotland’s care-homes from March 2020 to October 2021: national linked data cohort analysis

**DOI:** 10.1093/ageing/afae015

**Published:** 2024-02-10

**Authors:** Jennifer Kirsty Burton, Megan McMinn, James E Vaughan, Glenna Nightingale, Jacques Fleuriot, Bruce Guthrie

**Affiliations:** Academic Geriatric Medicine, School of Cardiovascular and Metabolic Health, College of Medical, Veterinary and Life Sciences, University of Glasgow, Glasgow Royal Infirmary, GlasgowG31 2ER, UK; Public Health Scotland, Glasgow G2 6QE, UK; Usher Institute, University of Edinburgh, Edinburgh EH8 9AG, UK; School of Informatics, University of Edinburgh, Edinburgh EH8 9AB, UK; Nursing Studies, School of Health in Social Science, University of Edinburgh, Edinburgh EH8 9AB, UK; Usher Institute, University of Edinburgh, Edinburgh EH8 9AG, UK; Advanced Care Research Centre, Usher Institute, University of Edinburgh, Edinburgh EH8 9AG, UK; Advanced Care Research Centre, Usher Institute, University of Edinburgh, Edinburgh EH8 9AG, UK

**Keywords:** long-term care, COVID-19, epidemiology, care-homes, data linkage, older people

## Abstract

**Background:**

The impact of the COVID-19 pandemic on long-term care residents remains of wide interest, but most analyses focus on the initial wave of infections.

**Objective:**

To examine change over time in: (i) The size, duration, classification and pattern of care-home outbreaks of COVID-19 and associated mortality and (ii) characteristics associated with an outbreak.

**Design:**

Retrospective observational cohort study using routinely-collected data.

**Setting:**

All adult care-homes in Scotland (1,092 homes, 41,299 places).

**Methods:**

Analysis was undertaken at care-home level, over three periods. Period (P)1 01/03/2020-31/08/2020; P2 01/09/2020-31/05/2021 and P3 01/06/2021–31/10/2021. Outcomes were the presence and characteristics of outbreaks and mortality within the care-home. Cluster analysis was used to compare the pattern of outbreaks. Logistic regression examined care-home characteristics associated with outbreaks.

**Results:**

In total 296 (27.1%) care-homes had one outbreak, 220 (20.1%) had two, 91 (8.3%) had three, and 68 (6.2%) had four or more. There were 1,313 outbreaks involving residents: 431 outbreaks in P1, 559 in P2 and 323 in P3. The COVID-19 mortality rate per 1,000 beds fell from 45.8 in P1, to 29.3 in P2, and 3.5 in P3. Larger care-homes were much more likely to have an outbreak, but associations between size and outbreaks were weaker in later periods.

**Conclusions:**

COVID-19 mitigation measures appear to have been beneficial, although the impact on residents remained severe until early 2021. Care-home residents, staff, relatives and providers are critical groups for consideration and involvement in future pandemic planning.

## Key Points

- The impact of the COVID-19 pandemic on long-term care residents remains of public, professional and political interest.
- The impact of COVID-19 on residents remained severe until early 2021 where significantly reduced mortality is observed.
- Larger care-homes continued to be at increased risk of experiencing outbreaks, but the association weakened in later periods.
- Future pandemic planning must incorporate learning from COVID-19 and provide contextually appropriate protections for citizens living in long-term care.
- A data-informed response requires prioritising the routine identification of the care-home population in national data collection.

## Background

Long-term care residents globally have been the group most affected by the COVID-19 pandemic [1], with residents accounting for 20, 25 and 33% of COVID-19 deaths in Wales, England and Scotland, respectively [2]. Pandemic care-home experiences remain the subject of significant public and policy interest, and are a focus for ongoing public inquiries [3]. However, the impact remains under-researched, with most research restricted to the initial wave of infections [4–10]. While pandemic epidemiology has been examined for the whole population of Scotland [11], care-home epidemiology has not been considered beyond June 2020 [12, 13]. Analyses extending to March 2021 in England have been limited to mortality, finding significant increases in the first two waves, although excess mortality compared to over 65s living at home was only detected in the first wave [14]. In Wales, longitudinal cohort analyses has examined risk factors for infections, including age, community prevalence and hospital admission [15, 16]. Internationally, variables such as neighbourhood socio-economic position, occupancy, ownership and crowding have been examined [6, 17–19]. Although international comparisons are complicated by structural and contextual differences in the configuration of care services [20], large care-home outbreaks are a common feature of periods whenever COVID community incidence is high [21].

Understanding the impact of the pandemic beyond the initial wave is critical to understand the effectiveness of additional protection measures for care-homes deployed later. These included improved guidance and access to personal protective equipment, routine testing, and improvement to structural factors such as sick pay for staff, reduced working in multiple homes and staff cohorting in outbreaks [22, 23]. Vaccination is highly effective at the individual level but the impact on care-home outbreaks and outcomes is uncertain. Our aim was to understand changing impact over time of COVID-19 using national linked datasets to examine (i) The size, duration, classification and pattern of care-home outbreaks and mortality and (ii) care-home characteristics associated with an outbreak.

## Methods

### Population, data sources and record linkage

Adult care-homes in Scotland are 24-hour registered care facilities (registered with the Care Inspectorate) [24], some of which include the provision of on-site nursing care. Most provide services for older adults or those with learning disabilities, with smaller numbers for people with physical and sensory impairment or substance misuse [25].

Linked datasets used for this analysis included care-home characteristics, occupancy, case mix, COVID-19 tests and mortality (Supplementary Box 1). Data were linked using the national health service (NHS) Scotland unique identifier, the Community Health Index (CHI) number. As there is no reliable national data to identify those who live in or work in care-homes [26, 27], all positive tests and mortality data were manually reviewed using clinical information, linkage to CHI and address matching to assign cases and deaths to specific care-homes (see Supplementary Extended Methods).

### Time periods

We defined three periods, to account for the changing denominator of open care-homes, based on Care Inspectorate data. These were:

- Period One (P1): 1 March 2020 to 31 August 2020—start of the first wave of infections to a period of very low community transmission;
- Period Two (P2): 1 September 2020 to 31 May 2021—second wave of infections to completion of primary vaccination of residents and staff;
- Period Three (P3): 1 June 2021 to 31 October 2021—initial post-vaccination period with high community transmission.

Supplementary Box 2 provides a summary of key national changes in support for care-homes, including the timing of mitigation measures.

### Outcomes

The primary outcomes were the presence and characteristics of outbreaks of COVID-19. An outbreak was defined as a single positive polymerase chain reaction (PCR) test occurring in a resident either tested in the care-home or in the first 2 days after hospitalisation. Tests were only included if they were a first infection (no positive test in the preceding 90 days) or reinfection (≥90 days between sequential positive tests) [28]).

Outbreak characteristics were size, duration and classification. Size was the number of resident cases within an outbreak. An outbreak was considered to have ended if there were no further new cases among residents for a period of 28 days. Duration was measured in days from the first case to the end of outbreak. Outbreaks were classified into: resident cases only; resident cases with staff cases in the 7 days before the first resident case; resident cases with staff cases detected in both the previous 7 days and the following 14 days; and resident cases with subsequent staff cases in the following 14 days.

COVID-19 mortality was defined as the presence of ICD-10 codes U071/U072 as the underlying cause of death, and COVID-19 associated mortality as U071/U072 recorded elsewhere on the death certificate.

### Statistical analyses

Agglomerative cluster analysis of care-homes with an outbreak was used to compare the pattern of outbreaks in P1 and P2, using the first outbreak in each period. Analysis was based on clinically meaningful variables: number of beds, start month, outbreak duration, cases per bed, and deaths per bed. Full cluster methods are included in Supplementary Extended Methods.

Logistic regression was used to examine care-home characteristics associated with the presence of an outbreak. Care-home characteristics were defined immediately before each period. The presence of collinearity was checked by calculating the variance inflation factor. A single adjusted model was run for each study period. All potential predictors relevant to estimating odds of a care-home experiencing an outbreak were included in the adjusted model. Interactions were not investigated. Sensitivity analysis was undertaken including older adult care-home services only (based on service registration) [24].

## Results

Of 715,096 positive PCR tests for severe acute respiratory syndrome coronavirus 2 (SARS-CoV-2) obtained in Scotland between 1 March 2020 and 31 October 2021, a total of 17,480 eligible positive tests for COVID-19 were identified in care-home residents and staff. Care-home location could be assigned to 17,185 (98.3%) tests, of which 10,141 (59.0%) were in residents. P1 contributed 3,917 (22.8%), P2 contributed 9,909 (57.7%) and P3 contributed 3,359 (19.5%) combined resident and staff positive cases. Only 503 staff cases were identified in P1, compared to 4,568 in P2 and 1,973 in P3.

### Outbreaks over the three periods

**Figure 1:**
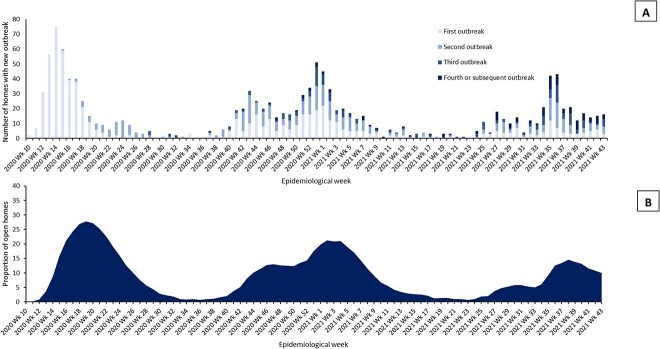
Number (A) of care-homes with new outbreak of COVID-19 by week and outbreak number and Proportion (B) of open care-homes with an ongoing COVID-19 outbreak by week (outbreak starts on first resident positive test and ends 28-days after last positive resident test)

There were 1,313 outbreaks involving residents. Of the 1,092 homes open at any point: 417 (38.2%) had no outbreaks; 296 (27.1%) had one, 220 (20.1%) had two, 91 (8.3%) had three and 68 (6.2%) had four or more (Figure 1A). The proportion of homes with an ongoing outbreak per week is summarised in Figure 1B, taking account of outbreak duration. In P1, 292 homes (26.9%) had one outbreak and 66 homes (6.1%) had two or more (Supplementary Table 1). In P2, 349 homes (32.4%) had one outbreak and 99 homes (9.2%) had two or more. In P3, 251 homes (23.5%) had one outbreak and 35 homes (3.3%) had two or more.

Of the 448 homes experiencing an outbreak in P2, 214 (47.8%) had experienced an outbreak in P1. Of the 286 homes experiencing an outbreak in P3, 125 (43.7%) had experienced an outbreak in P1, 162 (56.6%) had experienced an outbreak in P2 (84 [29.4%] had experienced outbreaks in both periods). In total, 45 outbreaks with 1–44 resident cases (mean 5.2) occurring in 42 care-homes included one or more reinfections. The earliest observed outbreaks involving reinfections occurred in December 2020, and one to three cases per outbreak were reinfections (mean 1.1).

### Outbreak size and duration

In P1, there were 431 outbreaks, involving 1–65 residents (mean 8 [SD 10] per outbreak; median 4 [IQR 10]). There was only one resident case detected in 150 of these outbreaks (34.8%). Duration of outbreaks ranged from 29 to 126 days (mean 45 days [SD 18.7]; median 40 [IQR 28]). In 162 (37.8%) outbreaks all positive tests were identified on the same day.

In P2, there were 559 outbreaks, involving 1–74 residents (mean 10 [SD 12.6] per outbreak; median 3 [IQR 13]). There was only one resident case detected in 219 (39.2%) of these outbreaks. Outbreak duration ranged from 29 to 125 days (mean 40 days [SD 14.9]; median 33 [IQR 20]). In 244 (43.6%) outbreaks all positive tests were identified on the same day.

In P3, there were 323 outbreaks, involving 1–32 residents (mean 4 [SD 5.3] per outbreak; median 2 [IQR 5]). There was only one resident case detected in 146 (45.2%) of these outbreaks. Outbreak duration ranged from 29 to 78 days (mean 34 days [SD 9.0]; median 29 [IQR 7]). In 177 (54.8%) outbreaks, all positive tests were identified on the same day.

### Outbreak classification

**Figure 2:**
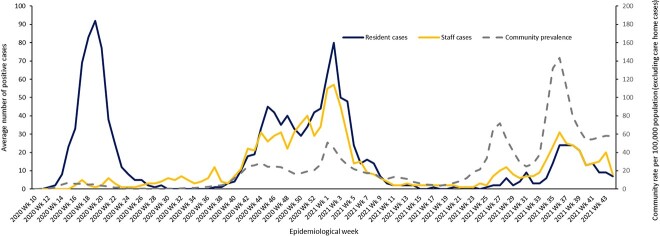
Seven-day rolling average of new positive cases of COVID-19 among residents and staff seven-day rolling average community prevalence per 100,000 population (excluding care-home cases)

The weekly rolling average of new positive cases among residents and staff is summarised in Figure 2. Almost two-thirds of positive staff tests (4,465, 63%) were not associated with a resident outbreak (i.e. occurred >7 days before the first resident case or > 14 days after the last resident case).

In P1, 402 (93.3%) outbreaks involved residents only. Eleven resident outbreaks (2.6%) were preceded by staff cases in the seven days before (seven of which also had staff cases detected in the following 14 days). Eighteen resident outbreaks (5.8%) only had positive staff cases detected in the 14 days after the resident outbreak started.

In P2, 242 (43.3%) outbreaks involved residents only. Two hundred and seven outbreaks (37.0%) were preceded by staff cases 7 days before (146 of which also had staff cases detected in the following 14 days). One hundred and ten resident outbreaks (19.7%) only had positive staff cases detected in the 14 days after the resident outbreak started.

In P3, 137 (42.4%) outbreaks involved residents only. One hundred and twenty-two outbreaks (37.8%) were preceded by staff cases in the 7 days before (77 of which also had staff cases detected in the following 14 days). Sixty-four resident outbreaks (19.8%) only had positive staff cases detected in the 14 days after the resident outbreak started.

### Resident mortality

**Figure 3:**
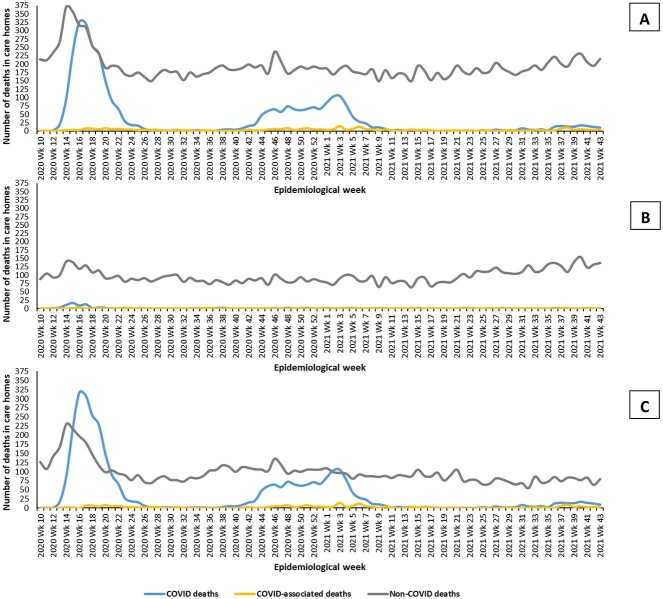
(A) Numbers of deaths by cause in all care-homes by week of death. (B) Numbers of deaths by cause in all care-homes by week of death in homes without an outbreak in the period (C) Numbers of deaths by cause in all care-homes by week of death in homes with an outbreak in the period

There were 20,352 deaths in 864 care-homes between 01/03/2020–31/10/2021. In P1, 815 (75.2%) open homes recorded any deaths, compared to 826 (76.6%) in P2 and 770 (72.2%) in P3. Across the whole study, 16,891 (83.0%) deaths were due to non-COVID-19 causes, 3,217 (15.8%) had COVID-19 recorded as the underlying cause of death, and 244 (1.2%) were COVID-19 associated deaths (Figure 3A). The COVID-19 mortality rate per 1,000 beds fell from 45.8 in P1, to 29.3 in P2, and 3.5 in P3 (Supplementary Figure 1).

Of the 3,461 care-home deaths with any mention of COVID-19, 2,420 (69.9%) had a positive PCR test before death (in P1 when testing was limited, 51.1% of COVID-19 deaths were preceded by a positive test versus 94.5% and 95.9% in P2 and P3). The median time to death after a positive test was 11 days (IQR 4). For COVID-19 associated mortality the median time to death was 22 days (IQR 34.5)) (test data available for 219 of 244 cases, 89.8%). Where COVID-19 was the underlying cause of death the median time to death was 11 days (IQR 9) (test data for 2,201 of 3,217 cases, 68.4%).

In P1 and P2, more deaths overall occurred in homes which experienced outbreaks than homes without outbreaks (P1 4,874 versus 2,661; P2 5,136 versus 3,257 deaths in outbreak versus non-outbreak homes respectively). As a smaller proportion of homes experienced outbreaks of COVID-19 in P3, more deaths occurred in homes without an outbreak than those with one (1,795 versus 2,624) (Figure 3B and C). Almost all COVID-19 deaths occurred in care-homes with a known outbreak (defined as a resident with a positive PCR test), with 96.6% of COVID-19 deaths in P1 being in care-homes with an outbreak, compared to 98.8% in P2 and 95.1% in P3. In P1, 37.2% of all deaths in care-homes with an outbreak were due to COVID-19 compared to 2.4% in care-homes without an outbreak. The corresponding figures were 23.0% versus 0.4% in P2, and 7.6% versus 0.3% in P3.

### Outbreak pattern

Including 358 first outbreaks in P1 and 448 first outbreaks in P2, cluster analysis found three clusters with similar patterns in both periods labelled as ‘contained’ (clusters 1-A and 2-D; number denotes period), ‘severe’ (1-B and 2-E), and ‘severe in larger care-homes’ (1-C and 2-F) (Supplementary Table 2). Contained outbreaks in both periods were shorter and had fewer cases and deaths per bed than more severe outbreaks, but mortality in contained outbreaks in P2 (mean 0.01 deaths/bed) was lower than in P1 (mean 0.03 deaths/bed) despite similar outbreak durations and cases per bed. Severe outbreaks in P2 were of similar duration to P1 (mean 59 days versus 54 days) and had more cases per bed (mean 0.6 versus 0.3) but similar deaths per bed (mean 0.2 in both periods). Severe outbreaks in larger care-homes were of shorter duration in P2 (mean 47 days versus 71 days) and had similar cases per bed (mean 0.2 in both periods) but fewer deaths per bed (mean 0.04 versus 0.1).

**Figure 4:**
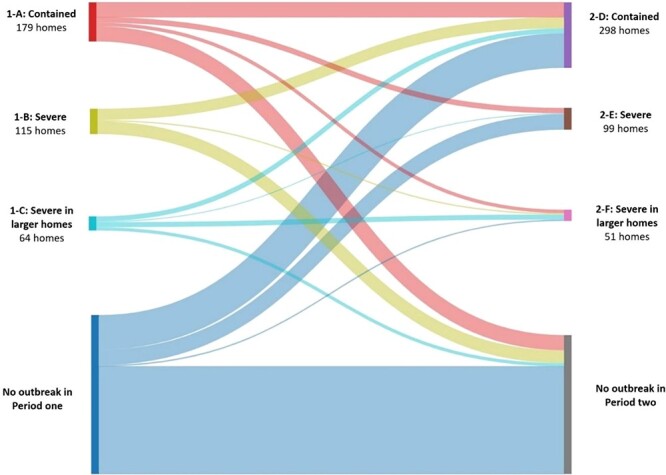
Outbreak experience of 1,077 homes open in periods one and two in terms of outbreak type. Footnotes. Period 1-A: Contained outbreak: average duration 36 days, 3 cases, 2 deaths. Period 2-D: Contained outbreak: average duration 34 days, 4 cases, 1 death. Period 1-B: Severe outbreak: average duration 54 days, 17 cases, 8 deaths. Period 2-E: Severe outbreak: average duration 59 days, 27 cases, 8 deaths. Period 1-C: Severe in larger homes: average duration 71 days, 14 cases, 8 deaths. Period 2-F: Severe in larger homes: average duration 47 days, 17 cases, 4 deaths. Complete data presented in Supplementary Table 2

In P1, 179 care-homes had a contained outbreak (1-A; number denotes period) compared to 298 in P2 (2-D) (Figure 4). Severe outbreaks occurred in 115 care-homes in P1 (1-B), compared to 99 in P2 (2-E). Severe outbreaks in larger care-homes were experienced by 64 care-homes in P1 (1-C) and 51 in P2 (2-F). Most care-homes experiencing an outbreak in P1 had the same type or less severe/no outbreak in P2, with most severe outbreaks in P2 (2-E or 2-F) being in care-homes without a P1 outbreak.

### Factors associated with outbreaks

Larger care-homes were much more likely to have an outbreak in all three periods, but associations were somewhat weaker in P2 and P3 (for ≥80 beds versus <20, adjusted OR [aOR] 49.6 [95%CI 17.8–150.6] in P1, 10.6 [95%CI 4.6–25.5] in P2, and 17.4 [95%CI 7.2–43.3] in P3) (Table 1). Other adult services and learning disabilities services were less likely to experience an outbreak in all three periods (aORs varying between 0.2 and 0.4).

**Table 1:** Care-home characteristics associated with having an outbreak of COVID-19

Care-home characteristic	Period One			Period Two			Period Three		
	Number (%) of homes with an outbreak	Univariate Odds Ratio 95% Confidence Interval	Adjusted Odds Ratio 95% Confidence Interval	Number (%) of homes with an outbreak	Univariate Odds Ratio 95% Confidence Interval	Adjusted Odds Ratio 95% Confidence Interval	Number (%) of homes with an outbreak	Univariate Odds Ratio 95% Confidence Interval	Adjusted Odds Ratio 95% Confidence Interval
Care-home size <20 places 20–29 places 30–39 places 40–49 places 50–59 places 60–69 places 70–79 places ≥80 places	11 (3.7) 32 (19.9) 48 (30.2) 62 (38.8) 36 (46.8) 68 (62.4) 30 (81.1) 71 (85.5)	REF 6**.5 (3.3–13.8)** **11.3 (5.9–23.6)** **16.5 (8.7–34.3)** **22.9 (11.1–50.6)** **43.3 (21.9–92.8)** **111.8 (42.7–333.8)** **154.4 (68.5–384.6)**	REF 3**.2 (1.4–8.0)** **5.3 (2.3–13.1)** **7.9 (3.4–19.8)** **9.3 (3.6–25.3)** **15.3 (6.2–40.5)** **37.4 (11.8–132.8)** **49.6 (17.8–150.6)**	40 (13.7) 51 (32.3) 68 (42.8) 81 (50.6) 48 (61.5) 68 (61.3) 26 (72.2) 66 (79.5)	REF 3**.0 (1.9–4.9)** **4.7 (3.0–7.5)** **6.5 (4.1–10.3)** **10.1 (5.8–18.0)** **10.0 (6.1–16.8)** **16.4 (7.6–38.3)** **24.6 (13.4–47.3)**	REF 2**.0 (1.1–3.7)** **3.3 (1.8–6.0)** **4.0 (2.1–7.7)** **5.9 (2.8–12.7)** **5.5 (2.7–11.5)** **7.6 (2.9–21.0)** **10.6 (4.6–25.5)**	22 (7.7) 34 (21.9) 45 (28.5) 53 (33.5) 29 (36.7) 39 (35.5) 17 (47.2) 47 (56.6)	REF 3**.4 (1.9–6.1)** **4.8 (2.8–8.5)** **6.1 (3.6–10.7)** **7.0 (3.7–13.3)** **6.6 (3.7–12.0)** **10.8 (4.9–23.9)** **15.7 (8.6–29.6)**	REF 3**.4 (1.7–6.9)** **4.5 (2.2–9.5)** **6.2 (3.0–13.5)** **7.1 (3.0–17.2)** **6.8 (3.0–16.0)** **12.6 (4.6–35.5)** **17.4 (7.2–43.3)**
Service type Older adult Other adult service^a^ Learning disabilities	346 (42.4) 6 (5.4) 6 (3.8)	REF **0.1 (0.0–0.2)** **0.1 (0.0–0.1)**	REF **0.2 (0.1–0.5)** 0.4 (0.1–1.0)	405 (49.6) 23 (21.2) 20 (13.1)	REF **0.3 (0.2–0.4)** **0.2 (0.1–0.2)**	REF **0.4 (0.2–0.8)** **0.4 (0.2–0.8)**	257 (31.8) 12 (11.2) 17 (11.3)	REF **0.3 (0.1–0.5)** **0.3 (0.2–0.4)**	REF 0.7 (0.3–1.5) 0.9 (0.4–1.8)
Sector Private Voluntary/not for profit Local authority/NHS	280 (41.2) 34 (13.2) 44 (29.9)	REF **0.2 (0.1–0.3)** **0.6 (0.4–0.9)**	REF 1.1 (0.6–1.9) **2.5 (1.4–4.4)**	331 (48.9) 67 (26.5) 50 (33.8)	REF **0.4 (0.3–0.5)** **0.5 (0.4–0.8)**	REF 1.2 (0.8–2.0) 1.3 (0.8–2.1)	216 (32.0) 39 (15.7) 31 (21.8)	REF **0.4 (0.3–0.6)** **0.6 (0.4–0.9)**	REF 0.9 (0.6–1.6) 1.0 (0.6–1.9)
Duration of service^b^ 0–2 years 3–5 years 6–10 years 11–14 years 15–20 years	68 (51.5) 30 (41.7) 98 (39.8) 44 (31.9) 118 (23.8)	**3.4 (2.3–5.1)** **2.3 (1.4–3.8)** **2.1 (1.5–2.9)** 1.5 (1.0–2.3) REF	1.4 (0.8–1.9) 1.8 (0.9–3.4) 1.1 (0.7–1.8) 0.9 (0.5–1.6) REF	89 (55.6) 34 (50.0) 106 (45.1) 49 (39.2) 170 (34.7)	**2.4 (1.6–3.4)** **1.9 (1.1–3.1)** **1.5 (1.1–2.1)** 1.2 (0.8–1.8) REF	1.0 (0.6–1.6) 1.1 (0.6–1.9) 0.9 (0.6–1.4) 0.8 (0.5–1.3) REF	30 (25.9) 34 (34.3) 72 (30.1) 26 (23.2) 124 (24.8)	1.1 (0.7–1.7) 1.6 (1.0–2.5) 1.3 (0.9–1.8) 0.9 (0.6–1.5) REF	0.6 (0.4–1.1) 0.8 (0.5–1.4) 0.9 (0.6–1.3) 0.7 (0.4–1.2) REF
Risk Score^c^ Low risk Medium risk High risk	194 (28.2) 81 (36.3) 83 (47.7)	REF 1**.4 (1.1–2.0)** **2.3 (1.6–3.3)**	REF 0.8 (0.6–1.3) 1.2 (0.8–1.9)	134 (27.9) 101 (45.9) 208 (56.5)	REF 2**.2 (1.6–3.1)** **3.4 (2.5–4.5)**	REF 1.2 (0.8–1.8) 1.6 (1.1–2.3)	102 (19.8) 131 (34.4) 53 (31.7)	REF 2**.1 (1.6–2.9)** **1.9 (1.3–2.8)**	REF 1.2 (0.8–1.7) 0.8 (0.5–1.3)
Nursing care No nursing care Nursing care	71 (15.4) 282 (46.3)	REF 4**.7 (3.5–6.4)**	REF 1**.7 (1.1–2.7)**	108 (26.9) 305 (50.7)	REF 2**.8 (2.1–3.7)**	REF 0.8 (0.6–1.2)	75 (18.7) 202 (33.6)	REF 2**.2 (1.6–3.0)**	REF 0.9 (0.6–1.3)
Urban Rural^d^ Large Urban Areas Other Urban Areas Accessible Small Towns Remote Small Towns Accessible Rural Remote Rural	156 (48.3) 128 (33.7) 30 (30.9) 10 (15.2) 28 (22.4) 6 (6.5)	**13.5 (6.2–35.6)** **7.4 (3.4–19.3)** **6.5 (2.7–18.1)** 2.6 (0.9–8.0) **4.2 (1.8–11.6)** REF	**7.3 (2.9–21.2)** **3.2 (1.4–8.9)** **4.8 (1.8–14.2)** 1.9 (0.6–6.0) **3.4 (1.3–10.1)** REF	153 (47.7) 177 (46.8) 36 (37.5) 18 (27.3) 48 (38.4) 16 (17.4)	**4.3 (2.5–8.0)** **4.2 (2.4–7.7)** **2.9 (1.5–5.7)** 1.8 (0.8–3.9) **3.0 (1.6–5.8)** REF	1.6 (0.8–3.2) 1.9 (1.0–3.9) 2.0 (0.9–4.3) 1.6 (0.7–3.6) **2.4 (1.1–5.1)** REF	89 (29.3) 115 (30.7) 18 (18.8) 11 (16.9) 39 (31.0) 14 (15.6)	**2.1 (1.2–4.1)** **2.4 (1.3–4.6)** 1.3 (0.6–2.7) 1.1 (0.5–2.6) **2.4 (1.3–5.0)** REF	1.1 (0.5–2.5) 1.3 (0.6–2.6) 0.8 (0.4–1.9) 0.8 (0.3–2.0) 1.6 (0.7–3.6) REF
Community prevalence^e^ Per 100 cases per 100,000 population increase	NA	**1.4 (1.3–1.6)**	**1.2 (1.1–1.4)**	NA	**1.03 (1.02–1.04)**	**1.03 (1.02–1.04)**	NA	**1.02 (1.01–1.03)**	**1.01 (1.00–1.02)**
Outbreak in previous period(s) No prior outbreak Outbreak in P1 Outbreak in P2 Outbreak in P1 & P2	NA	NA	NA	234 (32.4) 214 (60.1) NA NA	REF 3**.1 (2.4–4.1)** NA NA	REF 0.9 (0.6–1.3) NA NA	83 (17.1) 41 (29.3) 78 (34.2) 84 (39.6)	REF 2**.0 (1.3–3.1)** **2.5 (1.8–3.6)** **3.2 (2.2–4.6)**	REF 0.7 (0.4–1.3) 1.3 (0.8–1.9) 0.9 (0.5–1.5)
Occupancy 0–89% 90–100%	NA	NA	NA	223 (44.2) 176 (42.7)	REF 1.0 (0.8–1.3)	REF 1**.5 (1.1–2.1)**	160 (27.5) 125 (27.4)	REF 1.0 (0.8–1.4)	REF 1.1 (0.8–1.5)
Residents with significant cognitive impairment <33% 34–66% 67–100%	NA	NA	NA	38 (28.6) 121 (54.3) 240 (42.9)	REF 2**.6 (1.7–4.2)** **2.0 (1.3–3.0)**	REF 1.5 (0.9–2.6) 1.2 (0.7–2.0)	30 (17.0) 68 (28.5) 186 (30.0)	REF 1**.8 (1.1–2.9)** **2.2 (1.4–3.4)**	REF 1.1 (0.7–1.8) 1.7 (1.0–2.7)

Adjusted Odds Ratio—all variables with estimates shown in the columns for each Period are contained within the full adjusted model for that period.

Bold text denotes statistically significant results.

^a^Adult care-homes for: Alcohol and Drug Misuse; Blood Borne Viruses; Mental Health Problems, Physical and Sensory Impairment; Respite and Short Breaks

^b^Duration of care-home service is years since registration of service;

^c^Risk Assessment Document Score based on Care Inspectorate inspections;

^d^Urban Rural Classification based on Scottish Government 2016 classification incorporating population and accessibility;

^e^Rate for full period in the Integration Authority, community tests—tests conducted in home. OR is per 100 increase in rate per 100,000; observed range of prevalence is 31 to 753 (Period One), 316 to 7,854 (Period Two) and 2,564 to 12,225 (Period Three) per 100,000.

In P1, provision of nursing care (aOR 1.7, 95%CI 1.1 to 2.7), local authority/NHS homes (aOR 2.5, 95%CI1.4–4.4) and more urban location (e.g. large urban area versus remote rural aOR 7.3 (2.9–21.2) were associated with outbreaks, but not in P2 or P3. The prevalence of COVID-19 in the local authority where the care-home was located was associated with outbreaks in all three periods, but with the strongest association in P1 (OR per 100 cases/100,000 population increase 1.2 95%CI 1.1–1.4). Higher bed occupancy could only be measured in P2 and P3, and was associated with outbreaks in P2 (aOR occupancy 90–100% versus <90% 1.5, 95%CI 1.1–2.1) but not in P3. Having an outbreak in a previous period was associated with outbreaks in univariate but not adjusted analysis. Other care-home characteristics which were associated with having an outbreak in univariate analysis were not consistently associated with outbreaks after accounting for care-home size (e.g. sector, duration of service, regulatory risk score and proportion of residents with significant cognitive impairment). Sensitivity analysis restricted to older adult care-homes was consistent with primary analysis (Supplementary Tables 3 and 4).

## Discussion

### Summary of findings

More than three-fifths of care-homes experienced at least one resident outbreak. Outbreaks were common in all three periods and frequently multiple, with 6.2% of homes having four or more outbreaks across the study. At the peak of P1, over a quarter of all homes in Scotland had an active outbreak, and over a fifth at the peak of P2. The size of outbreaks increased in the second period (at least partly due to better ascertainment) but considerably reduced in size in P3 after completion of primary vaccination. COVID-19 mortality declined across the three periods, although it remained high in P2, before dropping sharply in P3. COVID-19 deaths almost all happened in homes with known outbreaks, consistent with the outbreak definition being accurate. Almost all (93%) of residents with COVID-19 recorded on their death certificate were deemed to die from the virus (recorded as underlying cause of death), rather than with it (recorded elsewhere on the death certificate). In cluster analysis, three clusters were consistently observed in P1 and P2, but more outbreaks in P2 were ‘contained’ and fewer were ‘severe’, illustrating the changing outbreak experience over time. Although associations were somewhat weaker in P2 and P3, care-home size was very strongly associated with outbreaks. Having outbreaks in previous periods did not appear protective of outbreaks in subsequent periods.

### Strengths and limitations

A key strength is examination of outbreaks in all care-homes in a whole country over three important periods which are distinctive in the protective measures in place (Supplementary Box 1). Data are inclusive of all residents, using routinely-collected data, so not reliant on individual consent which is problematic in this setting [29]. We also included cases detected on the first 2 days of a hospital admission from a care-home, to improve outbreak ascertainment.

An important limitation is that our outbreak definition focuses on resident cases, because staff cases are under-ascertained, particularly during the first period, where staff testing was restricted and initially undertaken externally from the care-home at public test sites, unless Public Health Teams undertook mass testing. Only staff tests which can be associated with the care-home can be included in our analyses and data quality improves when care-homes were able to undertake testing themselves. Resident testing was also restricted in P1 meaning resident *cases* will be under-ascertained, although the mortality data is consistent with good ascertainment of outbreaks in P1 even if not all residents with infection were tested.

Although we have robust data on deaths occurring within the care-home, we are not able to reliably identify residents whose deaths occurred in hospital, reflecting the ongoing challenge of national data sources in identifying the care-home population [26].

Our focus was care-home level analysis, but additional insights could have been obtained with more in-depth resident characteristics, not systematically available in national data [30].

Finally, some care-home characteristics are likely to vary within periods, for example quality of service (measured by the proxy of regulatory risk Risk Assessment Document (RAD) score score) and occupancy, but timely data were not consistently available [30].

### Comparison with other literature

The P1 findings when few mitigations were in place are consistent with other studies of the first wave, finding high impact in terms of mortality in care-homes with outbreaks [31, 32]. The care-home characteristics associated with first wave outbreaks in previous studies are similar to those described elsewhere [33, 34], with care-home size dominating, although this association weakened somewhat in later periods. Changes in the epidemiology of outbreaks have also been reported in Northern Ireland, with more widespread testing and improved outbreak ascertainment, and evidence that vaccination reduced outbreak severity [35, 36]. Notably, there were still large numbers of outbreaks in P2 after the implementation of multiple protective measures including routine testing of all care-home admissions from late April 2020. COVID-19 entered care-homes *via* multiple routes [37], but these findings are therefore consistent with previous UK studies’ conclusions that hospital discharge of untested patients to care-homes was likely not the major driver of care-home outbreaks in P1 [12, 33, 38–40]. Consistent with evidence about the effectiveness of vaccines in residents at individual-level [41, 42], this study provides important evidence that the prioritisation of the care-home population for vaccination was associated with large reductions in the number, size and duration of outbreaks, and sharply reduced COVID-19 mortality.

### Implications

Accepting the limitations of observational data it appears that the protective measures put in place before P2 have some mitigating effects. Outbreaks were more spread out in time (which is important for avoiding overloading public health response), and there was some decrease in severity, although there were still numerous outbreaks with high mortality. In principle, some (but not all) of the harm in P1 was therefore likely preventable with more rapid deployment of protective measures, although the transmissibility of COVID-19, initial lack of treatments and the dominance of age [43] and comorbidity [44] as risk factors for severe disease meant that it was likely inevitable that many care-homes would experience outbreaks with increased mortality, as was observed in P2. The care sector was notably absent from pandemic planning, for example only appearing as a potential source of ‘surge capacity’ to support the NHS rather than how to protect residents [45]. A key lesson for future pandemic planning is that the care-home sector has to be actively considered to maximise protection for a significant group of highly-vulnerable citizens [46].

It remains problematic for current care and future pandemic management that identification in routine data of care-home residents and people in receipt of care-at-home is still unreliable in all four UK countries [26]. It is regrettable that none has committed to resolving this data gap, which makes some of the most vulnerable citizens largely invisible in routine data, contributing to their low priority in policy and planning.

There is a need to better understand the indirect impact of COVID-19 on care-home residents, families and friends, and staff. For example, control measures which had some effect in reducing the risk of transmission [47] are recognised to have adverse effects on individuals and relationships [48–50]. Research is needed to understand the balance of benefits and harms of different control measures [51]. Similarly, we need to understand the role of the built environment in reducing risk of infections [52]. A care-home is the resident’s home rather than a short-term clinical setting, and optimal implementation of infection prevention and control measures in this context requires further research.

## Supplementary Material

aa-23-1356-File002_afae015

## Data Availability

Requests for access to the data may be submitted to Public Health Scotland via the Public Benefit and Privacy Panel for Health and Social Care (https://www.informationgovernance. scot.nhs.uk/pbpphsc/). Source code to undertake analysis is available from the authors on reasonable request.
