# Supplementary material for: Analysis of the impact of COVID-19 on Scotland’s care-homes from March 2020 to October 2021: national linked data cohort analysis
Source: Age Ageing. 2024 Feb 10;53(2):afae015. doi: 10.1093/ageing/afae015 (PMC10859243; doi:10.1093/ageing/afae015)
Supplement: aa-23-1356-File002_afae015 [file aa-23-1356-file002_afae015.docx]

**Supplementary Materials**

|  | **Page** |
| --- | --- |
| **Supplementary Box 1: Datasets and variables used for analysis** | **2** |
| **Supplementary extended methods**  **Supplementary Box 2:** Summary of key changes in support for care homes in national pandemic response | **3**  **6** |
| **Supplementary Table 1:** Care home characteristics of services in Scotland per period | **7** |
| **Supplementary Table 2:** Agglomerative cluster analysis on the 1,077 homes open in both periods one and two | **8** |
| **Supplementary Table 3:** Care home characteristics of older adult services in Scotland per period | **9** |
| **Supplementary Table 4:** Care home characteristics of older adult services associated with having an outbreak of COVID-19 | **10** |
| **Supplementary Figure 1:** Mortality rate/1000 bed by cause in care homes with and without an outbreak in each period | **12** |
| **Reference list** | **13** |

**Supplementary Box 1: Datasets and variables used for analysis**

| **Care Inspectorate datastore:**(1) publicly available data describing adult care home services characteristics We used: number of registered places; service type (older adult, learning disability or other adult); sector providing care (private, voluntary/not for profit or Local authority/NHS); Risk Assessment Document (RAD) Score assigned by inspectors to determine frequency of regulatory oversight (low risk, medium risk, high risk);(2) and duration of care home service (years, calculated from registration year).  **Care Inspectorate Data & Intelligence Team:** supplied variable from Annual Return dataset on whether the home provided nursing care to residents (yes/no/missing)  **Community Health Index (CHI) Register:** extracts from August 2020, December 2020, April 2021 and August 2021 were made available to identify residential address, date of address change and presence of institution flag – used to allocate tests to specific care home locations  **Electronic Communication of Surveillance in Scotland (ECOSS):** dataset of all SARS-CoV-2 Polymerase Chain Reaction (PCR) tests performed in Scotland between 1 March 2020 and 31 October 2021. Positive test data used to identify outbreaks  **National Records of Scotland (NRS) death registration data:** including date of death, location of death and cause(s) of death from 1 March 2020 to 31 October 2021 used to identify deaths occurring in care homes in Scotland.  **Public Health Scotland Daily Case Trends by Integration Authority open data:**(3) daily cases for each local-authority area. The prevalence of COVID-19 in the community in each analysis period was estimated, after removing tests conducted in care homes.  **Scottish Government Urban/Rural Classification 2016 open data**:(4, 5) care home postcode was used to define location in terms of settlement size and distance from an urban centre (large urban areas, other urban areas, accessible small towns, remote small towns, accessible rural, remote rural).  **TURAS Care Management (Safety Huddle):**(6) data submitted by care homes from August 2020 onwards, used to identify occupancy (number of residents/number of registered places, expressed as a %) and the proportion of residents with significant cognitive impairment |
| --- |

**Extended methods**

*COVID Test Classification Methods*

There is no simple or single way of identifying tests conducted in care home residents or staff. Test methods and access to testing has varied during the pandemic, with routine access to testing for staff available from summer 2020. Flags and variables within routine data have evolved during the pandemic and are often applied retrospectively depending on clinical information provided. Additional work is required to allocate tests to specific care home locations, necessary to undertake outbreak analysis. Therefore, a multi-layered approach was taken to identify and classify tests to maximise ascertainment, using linked identifiable data in a secure research environment within Public Health Scotland. Tests were classified as resident or staff, and allocated to individual care home. If a care home location could not be identified, the test was not included.

A full extract of positive COVID tests from 1 March 2020 to 31 October 2021 was extracted from ECOSS.

This included 24,329 positive tests from 1 March to 30 June 2020; 52,585 positive tests from 1 July to 31 October 2020; 158,448 from 1 November 2020 to 28 February 2021; 92,809 from 1 March to 30 June 2021 and 386,925 from 1 July to 31 October 2021. The file was checked for presence of a CHI number, submitted address, submitted postcode and residence postcode (13,268 were unable to be matched to CHI; 125,257 had no submitted address; 4,759 had no submitted postcode; 14,235 had no residence postcode).

Positive tests were linked to CHI data to obtain individuals primary care registered address and, where applicable, when this address changed and if there is any institution code associated with their address. Positive test records were linked to CHI extracts as follows:

- March to June 2020 linked to February 2020 and August 2020 CHI
- July to October 2020 linked to August 2020 and December 2020 CHI
- November 2020 to February 2021 linked to December 2020 and April 2021 CHI
- March to June 2021 to April 2021 and August 2021 CHI
- July to October 2022 to August 2021 CHI.

Specific variables used in test adjudication were: ecoss_specimen_source_type (Residential care home); ecoss_category (Public Health Investigation (PHI) Care; Care Home Staff (CHS)); submitted_subject_address; submitted_subject_postcode; subject_residence_postcode; ecoss_address; ecoss_clinical_details; test_centre_group (care home); organisation_reference_ID; organisation_name; date_address_changed and institution code (93 or 98). The Unique Property Reference Number (UPRN) care home flag, created for the earlier stage of this work, was used for March to October 2020 test classification.

Comparison was made between submitted_subject_postcode and subject_residence_postcode to identify where these matched or varied to provide additional information for manual adjudication.

Records underwent manual review based on these variables, comparing them to registered adult care home services in Scotland, defined by the Care Inspectorate.

Tests were classified into residents or staff (with care home location attached); presumed staff (no care home identifiers); not adult care home; misclassified records (evidence suggesting test not for a care home resident or staff, despite labels on test file); those where individuals became residents but tests were performed before their move into care and those which could not be classified (typically restricted to those without CHI numbers of care home identifiers). A total of 20,356 records were reviewed in this process.

For the remaining 670,524 records, comparison was made between submitted_subject_postcode and care home postcodes for adult care home services in Scotland. Manual review was undertaken of all test records matching a care home postcode to identify any tests missed by the above approach. In total 296 positive test records were identified as care home tests out of 4,218 postcode matches.

A file was created which included: CHI number, CH serial number (assigned for project), allocation (staff/resident), personal identifiers, test result, healthboard of test and specimen date.

Additional checks were made on the file to ensure individuals were classified as residents or staff (not as both) and to check the age range of participants. Records of children aged <14 years were excluded from staff data, often identified as children of staff in test clinical details. A total of 295 tests were excluded, of which 272 (92%) were undertaken on day 3 or more of a hospital admission, 16 tests were excluded as it was unclear if from residents or staff, and seven tests were excluded as aged under twelve so presumed to be children of staff.

Where CHI was missing from the test data, these were assumed to be unique individuals and infections. Reinfections were classified as those occurring 90 days after the last previous positive test – necessary to account for repeated and serial testing of those in hospital who required two negative tests to be discharged from hospital throughout the study period. We note this definition aligns with the national approach taken by Public Health Scotland in identifying reinfections. However, would not advocate use in the later Omicron wave beyond our study period. Finally, the test file was de-duplicated, keeping an individual’s first positive test for the primary analysis and retaining reinfections as a discrete flagged group.

*Location of Death Classification Methods*

Records for all deaths in Scotland were extracted from the National Records of Scotland’s Vital Events statistical database for the period 01/03/2020 through to 05/11/2021. Records contained place of death (address and postcode), and an institution code.(7) These 5-character institution codes are designed to uniquely identify institutions including care homes, hospitals, GP Surgeries, prisons and schools. The institution code was mapped to the CSNumber and UPRN for each care home service open during the period of analysis. This used postcode matching and manual allocation, including where care homes shared postcodes or postcodes were incorrect. Some hospices have institution codes for care home services and these deaths were excluded from care home deaths. The institution code recorded on the death record can be prone to mistakes (typographic, using codes for closed institutions, assigned to nearby institutions). Therefore, manual checking was carried out by searching for care home name, address, and postcode within the death records. Following this, <1% of deaths were reassigned to a new institution code. A small number of care homes did not have an institution code assigned at the point of searching, and so deaths occurring in these were manually identified and reassigned.

*Cluster analysis methods*

Cluster analysis of care homes with an outbreak was used to compare the pattern of outbreaks in periods one and two, using the first outbreak in a care home in each period. Clustering was based on clinically meaningful variables: number of beds (care home size), start month of outbreak (integer value), outbreak duration (days), number of cases per bed, and number of COVID-19 deaths per bed. Start month was included as our earlier work demonstrated different patterns in outbreaks based on their timing, observing ‘early’ and ‘late’ outbreaks.(8) In the regression analysis, we examined community prevalence as a risk factor for care home outbreak, and in the cluster analysis we used time the outbreak started as a proxy for this.

Data were normalised to unit mean and variance before further processing to ensure features were weighted equally. The clusters were initially identified with K-means++ and Agglomerative clustering, using the Scikit-learn package in Python, with evaluation of cluster solutions using the gap statistic, silhouette coefficient, Calinski-Harabasz index, and Davies-Bouldin index.(9-11) There was no strictly optimal number of clusters across both methods and all measures, though in general fewer clusters performed better. Final cluster choice was based on the gap statistic which validates a cluster solution compared to one that could be found in random data. In both periods, the three-cluster solution identified by Agglomerative clustering scored best for the gap statistic, and this is the solution presented. Cluster analysis in both periods was presented using a Sankey diagram to facilitate visual presentation of complex data in an accessible and clinically useful way.(12)

**Supplementary Box 2: Summary of key changes in support for care homes in national pandemic response**

| **Before March 2020**   - Minimal consideration in prior UK-wide pandemic planning - Lack of community focused infection prevention and control services, resources & guidance including those specifically for care home settings   **Period One: 1^st^ March 2020 to 31^st^ August 2020**   - Limited and variable access to Personal Protective Equipment initially until regional hubs were established in mid-April - Fluid-resistant surgical mask wearing initially restricted to confirmed cases of COVID-19, revised late June to recommend care home staff wear throughout shift - Initial clinical guidance in March prioritised care homes remaining open to facilitate flow across the health and social care system - Resident isolation period of seven days (late March) extended to fourteen days (late April) - Initially limited and geographically variable access to COVID-19 testing unless residents were admitted to hospital. Testing in the home restricted to small numbers of symptomatic residents. May changed to all residents in homes with outbreaks - No COVID-19 testing of new admissions from hospital or the community or for residents returning from hospital until announcement by Cabinet Secretary on 26^th^ April, requiring two negative tests before hospital discharge - Variable support for outbreak management at a local level - New clinical oversight arrangements introduced mid-May - Revised Inspection criteria from regulator June 2020 - Financial support for staff when self-isolating introduced in late June - Daily data reporting via Turas Care Management Safety Huddle Tool from August   **Period Two: 1^st^ September 2020 to 31^st^ May 2021**   - Good access to Personal Protective Equipment - Routine twice weekly testing of care home staff (initially using PCR, latterly using a mix of lateral flow test and PCR) – initial delays in homes receiving results later resolved - Routine testing of all new admissions to the home and for residents returning from hospital - Testing for visitors introduced in mid-December 2020 - Recognition of non-specific and atypical presentation of infection among residents and access to testing within the care home to permit early identification of infection - Earlier and better support for outbreak management - Guidance to reduce use of agency staffing and limit working across outbreak/non-outbreak homes following root cause analysis in November 2020 - Additional funding provided by government for staffing, sickness absence pay, personal protective equipment and administrative costs from December 2020 - Publication of Scottish COVID-19 Care Home Infection Prevention and Control Addendum to provide care home specific IPC advice (December 2020) and development of Care Home National Infection Prevention and Control Manual (published May 2021) - Vaccination programme for residents and staff starts on 14^th^ December 2020, voluntary participation, first vaccinations December 2020 to February 2021; second vaccinations completed by end of period two - February 2021 Open with Care Guidance for Supporting Meaningful Contact in Care Homes published for implementation   **Period Three: 1^st^ June 2021 to 31^st^ October 2021**   - July 2021 care home staff twice weekly lateral flow testing no longer required to be undertaken on-site in the care home and can be undertaken elsewhere based on discretion of the home - August 2021 updates to Open with Care and Open for Care (guidance for visiting professionals) issued to recognise the national move ‘beyond level 0’ in terms of the Scottish Government’s national response aiming to normalise vising arrangements within and outwith the care home including enhanced communal and group activities - September 2021 provision for individuals living in care homes to have a named visitor is having to undergo a period of isolation within the home or during a controlled outbreak |
| --- |

**Supplementary Table 1: Care home characteristics of services in Scotland per period**

| **Care home characteristics** | **PERIOD ONE** | **PERIOD TWO** | **PERIOD THREE** |
| --- | --- | --- | --- |
|  | **Number of care homes**  **(% of total 1084 homes)** | **Number of care homes**  **(% of total 1078 homes)** | **Number of care homes**  **(% of total 1066 homes)** |
| **Care home size**^a^  <20 places  20-29 places  30-39 places  40-49 places  50-59 places  60-69 places  70-79 places  ≥80 places | 298 (27.5)  161 (14.9)  159 (14.7)  160 (14.8)  77 (7.1)  109 (10.1)  37 (3.4)  83 (7.7) | 293 (27.2)  158 (14.7)  159 (14.7)  160 (14.9)  78 (7.2)  111 (10.3)  36 (3.3)  83 (7.7) | 287 (26.9)  155 (14.5)  158 (14.8)  158 (14.8)  79 (7.4)  110 (10.3)  36 (3.4)  83 (7.8) |
| **Occupancy**  0-89%  90-100%  Missing | Data not available for Period One | 504 (46.8)  412 (38.2)  162 (15.0) | 581 (54.5)  457 (42.9)  28 (2.6) |
| **Service type**  Older adult  Other adult service^b^  Learning disabilities | 817 (75.4)  111 (10.2)  156 (14.4) | 816 (75.7)  109 (10.1)  153 (14.2) | 808 (75.8)  107 (10.0)  151 (14.2) |
| **Sector**  Private  Voluntary/not for profit  Local authority/NHS | 680 (62.7)  257 (23.7)  147 (13.6) | 677 (62.8)  253 (23.5)  148 (13.7) | 676 (63.4)  248 (23.3)  142 (13.3) |
| **Duration of service^c^**  0-2 years  3-5 years  6-10 years  11-14 years  15-20 years | 132 (12.2)  72 (6.6)  246 (22.7)  138 (12.7)  496 (45.8) | 160 (14.8)  68 (6.3)  235 (21.8)  125 (11.6)  490 (45.5) | 116 (10.9)  99 (9.3)  239 (22.4)  112 (10.5)  500 (46.9) |
| **Risk Assessment Score^d^**  Low risk  Medium risk  High risk  Missing | 687 (63.4)  223 (20.6)  174 (16.0)  0 | 480 (44.5)  220 (20.4)  368 (34.2)  10 (0.9) | 516 (48.4)  381 (35.7)  167 (15.7)  2 (0.2) |
| **Residents with significant cognitive impairment**  0-33%  34-66%  67-100%  Missing | Data not available for Period One | 133 (12.3)  223 (20.7)  560 (51.9)  162 (15.0) | 176 (16.5)  239 (22.4)  620 (58.2)  31 (2.9) |
| **Nursing care**  No nursing care  Nursing care  Missing | 462 (42.6)  609 (56.2)  13 (1.2) | 401 (37.2)  602 (55.8)  75 (7.0) | 401 (37.6)  602 (56.5)  63 (5.9) |
| **Urban/Rural^e^**  Large Urban Areas  Other Urban Areas  Accessible Small Towns  Remote Small Towns  Accessible Rural  Remote Rural | 323 (29.8)  380 (35.1)  97 (8.9)  66 (6.1)  125 (11.5)  93 (8.6) | 321 (29.8)  378 (35.1)  96 (8.9)  66 (6.1)  125 (11.6)  92 (8.5) | 315 (29.5)  374 (35.1)  96 (9.0)  65 (6.1)  126 (11.8)  90 (8.4) |
| **Number of outbreaks^f^**  0 outbreaks  1 outbreak  ≥2 outbreaks | 726 (67.1)  292 (26.9)  66 (6.1) | 630 (58.4)  349 (32.4)  99 (9.2) | 780 (73.2)  251 (23.5)  35 (3.3) |

**Footnotes:**

a. Number of registered places,

b. Adult care homes for: Alcohol and Drug Misuse; Blood Borne Viruses; Mental Health Problems, Physical and Sensory Impairment; Respite and Short Breaks.

c. Duration of care home service is years since registration of service;

d. Risk Assessment Document Score based on Care Inspectorate inspections;

e. Urban Rural Classification based on Scottish Government 2016 classification incorporating population and accessibility;

f. A new outbreak was defined as a new resident positive test occurring 28 days or more after the last positive test in the home

**Supplementary Table 2: Agglomerative cluster analysis on the 1,077 homes open in both periods one and two**

| **Period** | **Cluster** | **Number of homes** | **Percentage of homes with outbreak** | **Percentage of open homes** | **Average number of beds** | **Average outbreak duration** | **Average number of cases** | **Cases per bed** | **Average number of deaths** | **Deaths per bed** |
| --- | --- | --- | --- | --- | --- | --- | --- | --- | --- | --- |
| **One** | 1-A: Contained | 179 | 50.0 | 16.6 | 54 | 36 days | 3 | 0.1 | 2 | 0.03 |
|  | 1-B: Severe | 115 | 32.1 | 10.7 | 48 | 54 days | 17 | 0.3 | 8 | 0.2 |
|  | 1-C: Severe in large homes | 64 | 17.9 | 5.9 | 87 | 71 days | 14 | 0.2 | 8 | 0.1 |
| **Two** | 2-D: Contained | 298 | 66.5 | 27.7 | 43 | 34 days | 4 | 0.1 | 1 | 0.01 |
|  | 2-E: Severe | 99 | 22.1 | 9.2 | 50 | 59 days | 27 | 0.6 | 8 | 0.2 |
|  | 2-F: Severe in large homes | 51 | 11.4 | 4.7 | 103 | 47 days | 17 | 0.2 | 4 | 0.04 |

**Supplementary Table 3: Care home characteristics of older adult services in Scotland per period**

| **Care home characteristics** | **PERIOD ONE** | **PERIOD TWO** | **PERIOD THREE** |
| --- | --- | --- | --- |
|  | **Number of care homes**  **(% of total 817 homes)** | **Number of care homes**  **(% of total 816 homes)** | **Number of care homes**  **(% of total 808 homes)** |
| **Care home size**^a^  <20 places  20-29 places  30-39 places  40-49 places  50-59 places  60-69 places  70-79 places  ≥80 places | 84 (10.3)  132 (16.2)  146 (17.9)  153 (18.7)  77 (9.4)  107 (13.1)  36 (4.4)  82 (10.0) | 83 (10.2)  130 (15.9)  145 (17.8)  153 (18.8)  78 (9.6)  110 (13.5)  35 (4.3)  82 (10.0) | 81 (10.0)  128 (15.8)  143 (17.7)  151 (18.7)  79 (9.8)  109 (13.5)  35 (4.3)  82 (10.1) |
| **Occupancy**  0-89%  90-100%  Missing | Data not available for Period 1 | 414 (50.7)  318 (39.0)  84 (10.3) | 460 (56.9)  338 (41.8)  10 (1.3) |
| **Sector**  Private  Voluntary/not for profit  Local authority/NHS | 606 (74.2)  90 (11.0)  121 (12.8) | 605 (74.1)  89 (10.9)  122 (14.9) | 605 (74.9)  84 (10.9)  119 (14.7) |
| **Duration of service^b^**  0-2 years  3-5 years  6-10 years  11-14 years  15-20 years | 115 (14.1)  56 (6.9)  211 (28.8)  114 (14.0)  321 (39.3) | 141 (17.3)  54 (6.6)  202 (24.8)  103 (12.6)  316 (38.7) | 99 (12.3)  82 (10.1)  208 (25.7)  92 (11.4)  327 (40.5) |
| **Risk Assessment Score^c^**  Low risk  Medium risk  High risk  Missing | 475 (58.1)  185 (22.6)  157 (19.2)  0 | 298 (36.5)  177 (21.7)  331 (40.6)  10 (1.2) | 329 (40.7)  322 (39.9)  155 (19.2)  2 (0.2) |
| **Residents with significant cognitive impairment**  0-33%  34-66%  67-100%  Missing | Data not available for Period 1 | 66 (8.1)  208 (25.5)  458 (56.1)  84 (10.3) | 85 (10.5)  224 (27.7)  489 (60.5)  10 (1.3) |
| **Nursing care**  No nursing care  Nursing care  Missing | 254 (31.1)  554 (67.8)  9 (1.1) | 214 (26.2)  548 (67.2)  54 (6.6) | 214 (26.5)  548 (67.8)  46 (5.7) |
| **Urban/Rural^d^**  Large Urban Areas  Other Urban Areas  Accessible Small Towns  Remote Small Towns  Accessible Rural  Remote Rural | 231 (28.3)  291 (35.6)  77 (9.4)  58 (7.1)  80 (9.8)  80 (9.8) | 231 (28.3)  291 (35.7)  77 (9.4)  58 (7.1)  80 (9.8)  79 (9.7) | 225 (27.8)  288 (35.6)  77 (9.5)  58 (7.2)  81 (10.0)  79 (9.8) |
| **Number of outbreaks^e^**  0 outbreaks  1 outbreak  ≥2 outbreaks | 471 (57.6)  280 (34.3)  66 (8.1) | 411 (50.4)  310 (38.0)  95 (11.6) | 551 (68.2)  223 (27.6)  34 (4.2) |

**Footnotes:**

a. Number of registered places,

b. Duration of care home service is years since registration of service;

c. Risk Assessment Document Score based on Care Inspectorate inspections;

d. Urban Rural Classification based on Scottish Government 2016 classification incorporating population and accessibility;

e. A new outbreak was defined as a new resident positive test occurring 28 days or more after the last positive test in the home

**Supplementary Table 4: Care home characteristics of older adult services associated with having an outbreak of COVID-19**

| **Care home characteristic** | **PERIOD ONE** | | | **PERIOD TWO** | | | **PERIOD THREE** | | |
| --- | --- | --- | --- | --- | --- | --- | --- | --- | --- |
|  | **Number (%) of homes with an outbreak** | **Univariate**  **Odds Ratio**  *95% Confidence Interval* | **Adjusted**  **Odds Ratio**  *95% Confidence Interval* | **Number (%) of homes with an outbreak** | **Univariate**  **Odds Ratio**  *95% Confidence Interval* | **Adjusted**  **Odds Ratio**  *95% Confidence Interval* | **Number (%) of homes with an outbreak** | **Univariate**  **Odds Ratio**  *95% Confidence Interval* | **Adjusted**  **Odds Ratio**  *95% Confidence Interval* |
| **Size**  <20 places  20-29 places  30-39 places  40-49 places  50-59 places  60-69 places  70-79 places  ≥80 places | 5 (6.0)  30 (22.7)  48 (32.9)  61 (39.9)  36 (46.8)  67 (62.6)  29 (80.6)  70 (85.4) | REF  **4.6 (1.9-14.1)**  **7.7 (3.2-23.1)**  **10.5 (4.4-31.1)**  **13.9 (5.5-42.8)**  **26.5 (10.8-80.2)**  **65.5 (21.0-248.8)**  **92.2 (33.6-307.5)** | REF  **3.1 (1.2-9.8)**  **5.4 (2.1-16.9)**  **7.8 (3.0-24.4)**  **9.0 (3.2-30.0)**  **14.1 (5.2-46.1)**  **30.8 (9.0-126.1)**  **44.0 (14.3-160.3)** | 15 (18.1)  44 (33.8)  62 (42.8)  78 (51.0)  48 (61.5)  68 (61.8)  25 (71.4)  65 (79.3) | REF  **2.3 (1.2-4.6)**  **3.4 (1.8-6.7)**  **4.7 (2.5-9.2)**  **7.3 (3.6-15.3)**  **7.3 (3.8-14.9)**  **11.3 (4.7-29.7)**  **17.3 (8.2-38.8)** | REF  1.6 (0.8-3.5)  **2.6 (1.3-5.5)**  **3.3 (1.6-7.1)**  **4.9 (2.1-11.6)**  **4.7 (2.1-11.0)**  **5.8 (2.0-17.4)**  **8.8 (3.5-23.3)** | 7 (8.6)  29 (22.7)  42 (29.4)  49 (32.5)  29 (36.7)  39 (35.8)  16 (45.7)  46 (56.1) | REF  **3.1 (1.4-8.0)**  **4.4 (2.0-11.2)**  **5.1 (2.3-12.8)**  **6.1 (2.6-16.2)**  **5.9 (2.6-15.2)**  **8.9 (3.3-26.2)**  **13.5 (5.9-35.5)** | REF  **2.7 (1.1-7.4)**  **3.9 (1.7-10.5)**  **4.7 (2.0-12.8)**  **5.6 (2.1-16.4)**  **5.4 (2.1-15.4)**  **9.5 (3.0-32.0)**  **13.0 (4.8-39.2)** |
| **Sector**  Private  Voluntary/not for profit  Local authority/NHS | 274 (45.2)  28 (31.1)  44 (36.4) | REF  **0.5 (0.3-0.9)**  0.7 (0.5-1.0) | REF  1.0 (0.5-1.8)  **2.4 (1.3-4.4)** | 313 (51.7)  44 (49.4)  48 (39.3) | REF  0.9 (0.6-1.4)  **0.6 (0.4-0.9)** | REF  1.3 (0.7-2.3)  1.3 (0.8-2.3) | 207 (34.2)  20 (23.8)  30 (25.2) | REF  **0.6 (0.3-1.0)**  **0.6 (0.4-1.0)** | REF  0.8 (0.4-1.4)  1.0 (0.6-1.7) |
| **Duration of service^a^**  0-2 years  3-5 years  6-10 years  11-14 years  15-20 years | 68 (59.1)  29 (51.8)  93 (44.1)  44 (38.6)  112 (34.9) | **2.7 (1.7-4.2)**  **2.0 (1.1-3.5)**  1.5 (1.0-2.1)  1.2 (0.7-1.8)  REF | 1.4 (0.8-2.5)  **1.9 (1.0-3.7)**  1.1 (0.7-1.6)  1.0 (0.6-1.7)  REF | 84 (59.6)  30 (55.6)  98 (48.5)  47 (45.6)  146 (46.2) | **1.7 (1.1-2.6)**  1.5 (0.8-2.6)  1.1 (0.8-1.6)  1.0 (0.6-1.5)  REF | 0.9 (0.5-1.5)  1.0 (0.5-2.0)  0.9 (0.6-1.3)  0.8 (0.5-1.4)  REF | 30 (30.3)  32 (39.0)  66 (31.7)  26 (28.3)  103 (31.5) | 0.9 (0.6-1.5)  1.4 (0.8-2.3)  1.0 (0.7-1.5)  0.9 (0.5-1.4)  REF | 0.7 (0.4-1.3)  0.9 (0.5-1.5)  0.8 (0.5-1.3)  0.7 (0.4-1.2)  REF |
| **Risk Score^b^**  Low risk  Medium risk  High risk | 185 (38.9)  80 (43.2)  81 (51.6) | REF  1.2 (0.8-1.7)  **1.7 (1.2-2.4)** | REF  0.9 (0.6-1.3)  1.2 (0.8-1.9) | 113 (37.9)  91 (51.4)  196 (59.2) | REF  **1.8 (1.2-2.6)**  **2.4 (1.8-3.3)** | REF  1.1 (0.7-1.7)  **1.4 (1.0-2.2)** | 84 (25.5)  122 (37.9)  51 (32.9) | REF  **1.8 (1.3-2.5)**  1.4 (0.9-2.2) | REF  1.2 (0.8-1.8)  0.8 (0.5-1.4) |
| **Nursing care**  No nursing care  Nursing care | 66 (26.0)  275 (49.6) | REF  **2.8 (2.0-3.9)** | REF  **1.6 (1.0-2.6)** | 81 (37.9)  294 (53.6) | REF  **1.9 (1.4-2.6)** | REF  0.9 (0.6-1.3) | 56 (26.2)  195 (35.6) | REF  **1.6 (1.1-2.2)** | REF  0.9 (0.6-1.4) |
| **Urban Rural^c^**  Large Urban Areas  Other Urban Areas  Accessible Small Towns  Remote Small Towns  Accessible Rural  Remote Rural | 152 (65.8)  127 (43.6)  28 (36.4)  10 (17.2)  23 (28.8)  6 (7.5) | **23.7 (10.7-63.3)**  **9.6 (4.4-25.2)**  **7.0 (2.9-20.0)**  2.6 (0.9-8.0)  **5.0 (2.0-14.2)**  REF | **7.9 (3.1-23.1)**  **3.3 (1.4-9.4)**  **4.4 (1.7-13.2)**  1.8 (0.6-6.0)  **2.8 (1.0-8.4)**  REF | 138 (59.7)  167 (57.4)  33 (42.9)  18 (31.0)  35 (43.8)  14 (17.7) | **6.9 (3.8-13.4)**  **6.3 (3.4-12.1)**  **3.5 (1.7-7.4)**  2.1 (0.9-4.7)  **3.6 (1.8-7.7)**  REF | 1.8 (0.9-4.1)  **2.3 (1.1-4.8)**  2.1 (0.9-4.9)  1.8 (0.7-4.3)  **2.2 (1.0-5.0)**  REF | 76 (33.8)  111 (38.5)  18 (23.4)  11 (19.0)  29 (35.8)  12 (15.2) | **2.8 (1.5-5.8)**  **3.5 (1.9-7.1)**  1.7 (0.8-3.9)  1.3 (0.5-3.2)  **3.1 (1.5-6.9)**  REF | 1.1 (0.5-2.6)  1.4 (0.7-3.2)  1.0 (0.4-2.4)  0.9 (0.4-2.4)  1.7 (0.7-4.0)  REF |
|  | **PERIOD ONE** | | | **PERIOD TWO** | | | **PERIOD THREE** | | |
| **Care home characteristic** | **Number (%) of homes with an outbreak** | **Univariate**  **Odds Ratio**  *95% Confidence Interval* | **Adjusted**  **Odds Ratio**  *95% Confidence Interval* | **Number (%) of homes with an outbreak** | **Univariate**  **Odds Ratio**  *95% Confidence Interval* | **Adjusted**  **Odds Ratio**  *95% Confidence Interval* | **Number (%) of homes with an outbreak** | **Univariate**  **Odds Ratio**  *95% Confidence Interval* | **Adjusted**  **Odds Ratio**  *95% Confidence Interval* |
| **Community prevalence^d^**  Per 100 cases per 100,000 population increase | NA | **1.6 (1.4-1.8)** | **1.2 (1.0-1.4)** | NA | **1.0 (1.0-1.1)** | **1.0 (1.0-1.0)** | NA | **1.0 (1.0-1.0)** | **1.0 (1.0-1.0)** |
| **Outbreak in previous period(s)**  No prior outbreak  Outbreak in P1  Outbreak in P2  Outbreak in P1 & P2 | NA | NA | NA | 196 (41.6)  209 (60.6)  NA  NA | REF  **2.2 (1.6-2.9)**  NA  NA | REF  0.9 (0.6-1.3)  NA  NA | 64 (23.2)  41 (30.6)  70 (36.6)  82 (39.6) | REF  1.5 (0.9-2.3)  **1.9 (1.3-2.9)**  **2.2 (1.5-3.2)** | REF  0.8 (0.4-1.3)  1.2 (0.8-1.9)  0.9 (0.5-1.5) |
| **Occupancy**  0-89%  90-100% | NA | NA | NA | 208 (50.2)  159 (50.0) | REF  1.1 (0.8-1.5) | REF  **1.6 (1.1-2.3)** | 146 (31.7)  110 (32.5) | REF  1.1 (0.8-1.5) | REF  1.2 (0.8-1.6) |
| **Residents with significant cognitive impairment**  <33%  34-66%  67-100% | NA | NA | NA | 29 (43.9)  115 (55.3)  223 (48.7) | REF  1.4 (0.8-2.4)  1.3 (0.8-2.2) | REF  1.3 (0.7-2.4)  1.0 (0.6-1.9) | 24 (28.2)  67 (29.9)  165 (33.7) | REF  1.0 (0.6-1.8)  1.3 (0.8-2.3) | REF  0.9 (0.5-1.7)  1.4 (0.8-2.5) |

Adjusted Odds Ratio – all variables with estimates shown in the columns for each Period are contained within the full adjusted model for that period

a. Duration of care home service is years since registration of service;

b. Risk Assessment Document Score based on Care Inspectorate inspections;

c. Urban Rural Classification based on Scottish Government 2016 classification incorporating population and accessibility;

d. Rate for full period in the Integration Authority, community tests – tests conducted in home. OR is per 100 increase in rate per 100,000; observed range of prevalence is 31 to 753 (Period One), 316 to 7854 (Period Two) and 2564 to 12225 (Period Three) per 100,000.

**Supplementary Figure 1: Mortality rate/1000 bed by cause in care homes with and without an outbreak in each period**

**
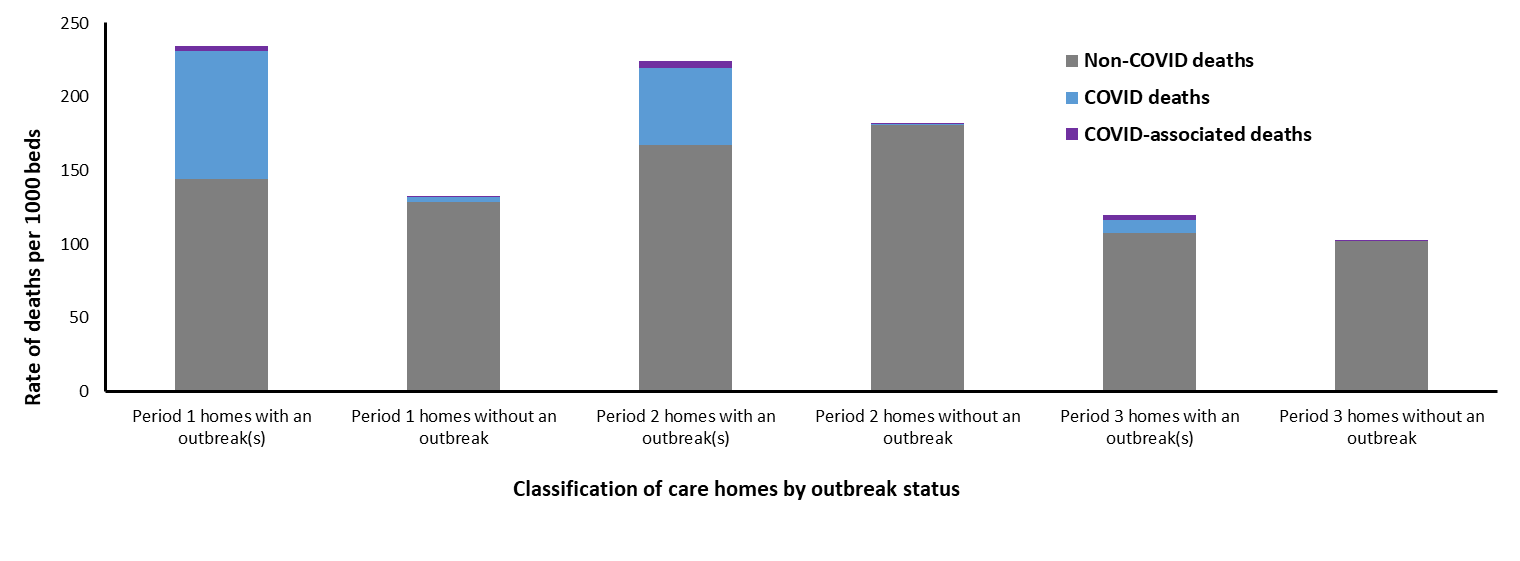
**

**Reference list**

1. Care Inspectorate. Datastore 2020 [cited 2020 14th October]. Available from: <https://www.careinspectorate.com/index.php/publications-statistics/93-public/datastore>.

2. Care Inspectorate. Risk Assessment 2020 [Available from: <https://www.careinspectorate.com/images/documents/148/Risk%20Assessment%20%E2%80%93%20Information%20for%20Care%20Service%20Providers.pdf>.

3. Public Health Scotland. Daily Case Trends By Council Area 2020 [cited 2020 26th October]. Available from: <https://www.opendata.nhs.scot/dataset/covid-19-in-scotland/resource/427f9a25-db22-4014-a3bc-893b68243055>.

4. Scottish Government. Scottish Government Urban Rural Classification 2016 [cited 2020 8th December]. Available from: <https://www2.gov.scot/Topics/Statistics/About/Methodology/UrbanRuralClassification>

5. National Records of Scotland. Scottish Postcode Directory Files 2020 [cited 2020 8th December]. Available from: <https://www.nrscotland.gov.uk/statistics-and-data/geography/our-products/scottish-postcode-directory/2020-2>

6. NHS Education for Scotland. TURAS Care Management 2020 [cited 2020 13th December]. Available from: <https://learn.nes.nhs.scot/34427/turas-care-management-user-guides>.

7. National Records of Scotland. Code-lists Used in Vital Event Statistics: Institutions 2022 [Available from: <https://www.nrscotland.gov.uk/files//statistics/vital-events/institution-codes-october-2021.xlsx>

8. Burton JK, McMinn M, Vaughan JE, Fleuriot J, Guthrie B. Care-home outbreaks of COVID-19 in Scotland March to May 2020: National linked data cohort analysis. Age and Ageing. 2021;50(5):1482-92.

9. Pedregosa F, Varoquaux G, Michel V, Thiron B, Grisel O, Blondel M, et al. Scikit-learn: Machine Learning in Python. Journal of Machine Learning Research. 2011;12:2825-30.

10. Ward Jr JH. Hierarchical grouping to optimize an objective function. Journal of the American Statistical Association. 1963;58:236-44.

11. Arthur D, Vassilvitskii S. K-means++ the advantages of careful seeding. Proceedings of the eighteenth annual ACM-SIAM symposium on Discrete algorithms. 2007:1027-35.

12. SankeyMATIC. Build a Sankey Diagram 2023 [Available from: <https://sankeymatic.com/build/>.
